# Prevalence of pharmacological and non-pharmacological coping mechanisms for anxiety management during the COVID-19 pandemic: investigating the transition to online learning among medical students

**DOI:** 10.1186/s12888-022-04372-6

**Published:** 2022-11-14

**Authors:** Firas K. Almarri, Ali M. Alaseem, Muteb S. Alanazi, Salman I. Alyahya, Naif H. Alsanad, Khalid E. Alswayed, Jowaher S. Alanazi, Tareq N. Alharby, Saleh A. Alghamdi

**Affiliations:** 1 College of Medicine, Imam Mohammad Ibn Saud Islamic University (IMSIU), Riyadh, Saudi Arabia; 2 Department of Otorhinolaryngology – Head and Neck Surgery, Ad Diriyah Hospital, Riyadh Third Health Cluster, Ministry of Health, Riyadh, Saudi Arabia; 3 Department of Pharmacology, College of Medicine, Imam Mohammad Ibn Saud Islamic University (IMSIU), Riyadh, Saudi Arabia; 4 Department of Clinical Pharmacy, College of Pharmacy, University of Hail, Hail, Saudi Arabia; 5 Department of Pharmacology and Toxicology, College of Pharmacy, University of Hail, Hail, Saudi Arabia; 6 Department of Clinical Neurosciences, College of Medicine, Imam Mohammad Ibn Saud Islamic University (IMSIU), Riyadh, Saudi Arabia

**Keywords:** Anxiety management, Copying mechanism, COVID-19 pandemic, Medical students, Online learning, Pharmacology

## Abstract

**Background:**

The coronavirus disease 2019 (COVID-19) pandemic had a devastating effect on college students worldwide. Here, the authors aimed to determine the prevalence of anxiety and its related coping strategies, provide a theoretical basis for understanding self-prescription, and identify the factors contributing to stress and anxiety in medical students during the pandemic.

**Methods:**

The authors conducted a cross-sectional study among medical students in Saudi Arabia from September to November 2020. They assessed anxiety using the GAD-7 scale based on seven core symptoms. The authors also examined perceived psychological stress using a single-item measure of stress, the factors contributing to stress during the transition to online learning and examinations, and related coping strategies. The Statistical Package for Social Sciences (SPSS) version 26.0 was used to examine the data for both descriptive and inferential analyses. Chi-square test, one-way ANOVA, and univariate linear regression were used to test the research hypotheses.

**Results:**

The authors collected and analyzed data from 7116 medical students distributed across 38 medical colleges. Among them, 40% reported moderate to severe anxiety symptoms. Pre-clinical and female students experienced more stress than clinical and male students. 12.19% (*n* = 868) of respondents reported using medication during their college years. Among those, 58.9% (*n* = 512) had moderate to severe anxiety, and the most commonly used drug was propranolol (45.4%, *n* = 394). Among the studied sample, 40.4% (*n* = 351) decreased their medication use after switching to online teaching. Most students used these medications during the final exam (35.8%, *n* = 311) and before the oral exam (35.5%, *n* = 308). In terms of coping strategies, males were much more likely to use substances than females, who mainly resorted to other strategies.

**Conclusions:**

This study provides a national overview of the impact of COVID-19 on the mental health of medical students. The results indicated that the pandemic is associated with highly significant levels of anxiety. These findings can provide theoretical evidence for the need for supportive psychological assistance from academic leaders in this regard.

**Supplementary Information:**

The online version contains supplementary material available at 10.1186/s12888-022-04372-6.

## Background

Students’ academic journey is filled with enormous obstacles, ranging from financial constraints to social issues to competitive academic challenges that may negatively affect their psychological well-being [1–3]. Factors such as increased academic workloads and student abuse (verbal, institutional, or physical abuse) might trigger psychological problems associated with stress and anxiety [4]. These factors can be interpreted and experienced as stressors when there is a demand-resource imbalance, a lack of control, and a lack of meaning [5]. Due to the intense and demanding nature of medical education, these three characteristics are heavily exacerbated among medical students, thereby putting them under tremendous pressure and subsequently negatively impacting their psychological resilience, which can precipitate anxiety [6]. A meta-analysis conducted by Quek et al. found that the global prevalence rate of anxiety among medical students (*n* = 40,348) is 33.8%, which is significantly higher than that in the general population [7].

The coronavirus disease 2019 (COVID-19) pandemic has potentially impacted medical students psychologically. The pandemic can plausibly be considered a mental health crisis, which has been confirmed by the current research on COVID-19. In a nationwide survey conducted by Qiu et al. [8], 35% of respondents (*n* = 52,730) across China experienced some form of psychological distress during the pandemic. A systematic review of studies analyzing the impact of the COVID-19 pandemic on mental health in the general population assessed anxiety symptoms in 11 of the 19 studies. It showed relatively high rates of association (up to 50.9%) with COVID-19 [9]. Furthermore, the COVID-19 pandemic has also necessitated significant changes in teaching style, thereby adding subsequent hurdles. The entire curriculum and examinations have shifted to a virtual online format. Therefore, attaining optimal academic performance goals has been dramatically challenged (especially for medical students), which might negatively influence psychological well-being [10]. The work of Cao et al., an assessment of the mental health of medical students (*n* = 7143), found that 24.9% (*n* = 1776) of respondents experienced anxiety associated with COVID-19 [11]. To cope with prevailing circumstances, students turned to various stress-relieving techniques, including pharmacological interventions. Studies of adopted coping strategies among medical students have shown the use of positive coping approaches such as positive reframing, planning, and religion; others established alcohol, tobacco, and drugs as strategies to cope with stress [12]. The use of drugs is a common technique to overcome anxiety disorder symptoms [13]. For instance, beta-blockers are commonly used to control blood pressure, including non-selective drugs such as propranolol, which has been shown to minimize performance anxiety [14]. In controlled double-blinded trials, propranolol showed favorable effects in the management of performance anxiety disorders [15–17]. Stress and anxiety can impair goal-directed attention and concentration, working memory, and perceptual-motor function, all of which are indispensable domains that enable medical students and physicians to provide safe and effective medical care to patients [18–20].

Students’ medical knowledge may also contribute to such self-diagnosis and self-treatment of anxiety. In Saudi Arabia, few studies have evaluated the use of propranolol among medical students. One study was conducted at King Saud bin Abdulaziz University for Health Sciences (KSAU-HS) in Riyadh and involved both medical and dental students. In the study, 30% (*n* = 100) of the 334 respondents reported using propranolol. Approximately 86% (*n* = 86) of the propranolol users did not have a proper clinical indication [21]. Another study conducted at King Saud University investigated the inappropriate use of beta-blockers in 22.4% (*n* = 198) of the 885 respondents and found that 13.9% (*n* = 123) self-prescribed the medication [22].

To date, the mental health of medical students and its related coping mechanisms, have received scant attention in the research literature, with far too little attention given to the pharmacological aspect of coping strategies. The pandemic has unleashed a global crisis with an unparalleled magnitude, posing unprecedented challenges to medical students who have been showing higher rates of suicidal ideation, increasing anxiety, depression, and stigmatization, and are less likely to seek psychological support [23]. In an attempt to safeguard the mental health of medical students, we aimed to determine the prevalence of anxiety and its coping mechanisms in the Kingdom of Saudi Arabia, before and during the transition to online or distance learning due to COVID-19. It is hoped that this research will contribute to a deeper understanding of factors affecting the national prevalence of anxiety, provide a theoretical basis for understanding self-prescription and off-label use of drugs, and identify contributing factors associated with stress and anxiety in medical students during the pandemic.

We hypothesize that (1) the overall negative psychological impact will be higher on clinical students and that (2) the mean levels of perceived psychological stress during the switch to online/distant learning are expected to be increased compared to levels of perceived stress during previous learning periods. We also predict that (3) the usage of anti-anxiety medications during the pandemic will be decreased compared to normal situations.

## Methodology

### Study design and participants

We conducted a cross-sectional study in Saudi Arabia during the pandemic from September to November 2020, using a standardized questionnaire, to assess the impact of COVID-19 and the transition to online synchronous learning on students’ mental health. During that time, the kingdom undertook strict measures to limit the spread of COVID-19, including a stay-at-home curfew; travel bans; closing down schools, universities, shopping malls, and mosques. As of 30th November 2020, there were ~ 357,623 confirmed cases and 5907 deaths [24]. A minimum sample size of 380 was calculated using the formula:

$$N=\frac{Z_{a/2}^2\times P\times \left(1-P\right)}{d^2}$$

An acceptable margin of error of 0.05 for the proportion was estimated at 95% confidence level. Based on the participants’ responses, it increased to 7116. Nonmedical students or medical students living outside of Saudi Arabia were excluded.

We utilized an online platform hosted by SurveyMonkey Inc. (San Mateo, California, USA; www.surveymonkey.com) to send the questionnaire along with a cover letter attached to a consent form. Participation was voluntary, with the option of withdrawing at any time. All responses were anonymous, with no e-mail addresses or ID information tracking. The Institutional Review Board (IRB) of Imam Mohammed Ibn Saud Islamic University (IMSIU) in Riyadh, Saudi Arabia, reviewed and approved this project (HAPO-01-R-011, Project No. 79–2020).

### Study measures

The questionnaire consisted of 19 items divided into six sections. In the first section, we included questions about demographic information, such as gender, medical class year, university, and region.

In the second section, we assessed anxiety levels using the generalized anxiety disorder scale (GAD-7) considering school suspension, online learning, and lockdown. The GAD-7 is an anxiety scale comprising seven items based on seven core symptoms with scores ranging from 0 to 3. The total scale score ranges from 0 to 21, with cut-off scores of 5, 10, and 15 indicating mild, moderate, and severe anxiety symptoms, respectively. The symptoms were reported by respondents using a 4-item Likert rating scale ranging from 0 (not at all) to 3 (almost every day). The GAD-7 scale is an efficient and sensitive tool to screen for anxiety and has shown excellent internal consistency (Cronbach’s *α* = .92) [25, 26].

In the third section, we examined perceived psychological stress using a single-item measure of stress [27] to determine the extent to which respondents experienced stress after transiting to online synchronous learning and their regular level of stress during traditional face-to-face learning. The responses were recorded using a 5-point Likert scale ranging from 0 (not at all) to 4 (to a very great extent).

In the fourth section, we investigated factors contributing to stress during the transition to online learning and examinations. After searching the literature and conducting informal interviews with multiple medical students from different class years, the research team yielded eight of the most reported items for respondents to choose from.

In the fifth section, we reported the history and pattern of anti-anxiety medication usage and its related characteristics during the traditional learning phase and the switch to online learning. A few questions about the usage and pattern of anti-anxiety medications were adapted from a previously validated questionnaire after obtaining the corresponding author’s permission [21]. Four main anti-anxiety medications were investigated: propranolol (Inderal®), benzodiazepine (Xanax®, Valium®), pregabalin (Lyrica®), and antidepressants (SSRI, TCA, and MOA).

In the final section, we utilized the brief coping orientation to problems experienced (Brief COPE) inventory [28] to determine the coping strategies adopted by respondents. The Brief COPE responses were scored from 1 to 4, ranging from “I have not been doing this at all” to “I have been doing this a lot.” The 24-item scores were averaged in pairs to produce 14 coping strategy scores. Linear regression modules for each coping strategy pair were fitted to estimate the differences in mean coping strategies by gender, medication usage, and medical class level with 95% confidence intervals (CIs).

To validate the questionnaire’s clarity, we piloted the final version of the questionnaire on 20 randomly selected medical students. These students were excluded from the final analysis. Face and content validity were established by three experts specializing in pharmacology, psychiatry, and medical education. After that, we further modified and updated the questionnaire for clarity and comprehensibility.

### Survey distribution

The survey was distributed nationally using a multifaceted approach. A list containing all 38 medical colleges (government and private) across all regions of Saudi Arabia was generated [the list is reported separately see Supplementary Table 1, Additional file 1]. As per the MOH in Saudi Arabia [29], the total number of enrolled medical students in 38 medical colleges for the academic year 2019–2020 is 32,696.

To ensure a successful distribution, a recruitment form was sent to data collectors (medical students) across all regions of Saudi Arabia through the e-mail addresses of each university. A total of 150 respondents out of 1250 were carefully chosen to participate in our study as data collectors. To facilitate normal distribution across all variables, we allocated four students from each university with equal gender and class level representation. A well-structured handbook with clear descriptions and instructions for the study was delivered to all data collectors. The data collectors then distributed the questionnaire to their colleagues through e-mails, social media platforms, or on-site distribution. We actively monitored the data collectors to ensure a representative sample from each region.

### Statistical analysis

We analyzed the data using IBM® Statistical Package for the Social Sciences (SPSS) version 26.0. We used descriptive statistics (e.g., frequencies, percentages, means, and standard deviations) to describe, summarize, and prepare the data analysis. We also used the chi-square test and one-way ANOVA to compare the groups and test for differences between different groups. We then conducted a univariate linear regression to determine whether gender, medication usage, and year of students significantly predicted coping strategies. The assumptions of the One-way ANOVA model were assessed to validate the procedure before performing the analysis. Normality was evaluated using the Shapiro- Wilk’s test; homoscedasticity was assessed using Levene’s test. We used a *p*-value < .05 and a 95% confidence interval to report the statistical significance and estimates in this study.

## Results

### Demographic characteristics of participants

**Fig. 1:**
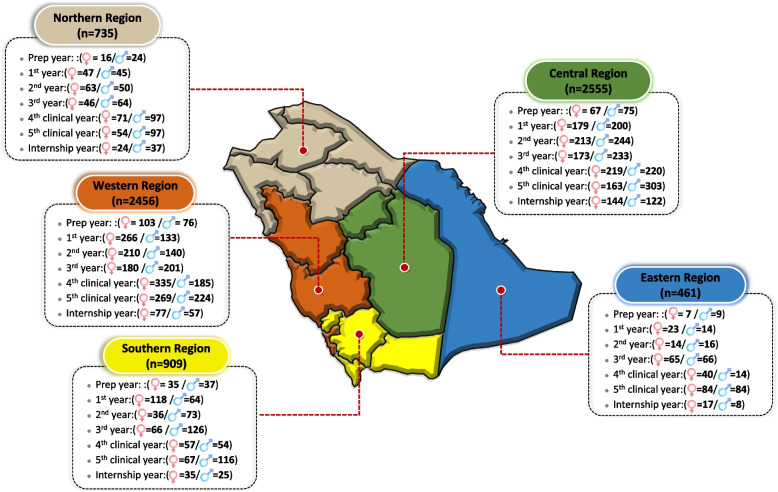
Demographic characteristics of 7116 medical students enrolled in all 38 medical colleges across all regions of Saudi Arabia in November 2020

Of the study population, 7116 out of 8865 medical students responded appropriately to all sections of the structured questionnaire, with a response rate of 80.27%. The sample was divided almost equally among genders, with 50.4% (*n* = 3583) being female and 49.6% (*n* = 3533) being male. Collectively, respondents from the first 4 years (pre-clinical years) constituted 53.6% (*n* = 3817) of the total sample, while students from the last 3 years (clinical years) constituted only 46.4% (*n* = 3299) (Fig. 1).

### Levels of anxiety among medical students during the pandemic and its association with medical class year and users of anti-anxiety medications

Of the 7116 medical students, approximately 28% (*n* = 2002) reported no symptoms of anxiety, whereas the proportion of students with mild, moderate, and severe anxiety was 31.4% (*n* = 2234), 24.5% (*n* = 1740), and 16% (*n* = 1140) respectively.

Of the 7116 students, 53.6% (*n* = 3817) were from preclinical years of study (first 4 years). Out of these preclinical students, 24.3% (*n* = 927) reported no anxiety symptoms, whereas 30.8% (*n* = 1176), 26.8% (*n* = 1023), and 18.1% (*n* = 691) reported mild, moderate, and severe anxiety symptoms, respectively. In contrast, 46.4% (*n* = 3299) were clinical students (last 3 years), out of which 32.6% (*n* = 1075) reported no anxiety symptoms, 32.1% (*n* = 1058), 21.7% (*n* = 717), and 13.6% (*n* = 449) reported mild, moderate, and severe anxiety symptoms, respectively.

Among the studied samples 12.2% (*n* = 868) of the students reported using medication throughout the course of their study. The most commonly used drugs were propranolol (45.39%, *n* = 394), followed by antidepressants (38%, *n* = 330), benzodiazepine (25.2%, *n* = 219), and pregabalin (10.9%, *n* = 95). About 16% (*n* = 139) of medication users had no anxiety, while 25% (*n* = 217), 32.9% (*n* = 286), and 26% (*n* = 226) had mild, moderate, and severe anxiety, respectively (Table 1).

**Table 1:** Descriptive statistics portraying differing anxiety levels of 7116 medical students across the Kingdom of Saudi Arabia according to the GAD-7-scale, sub-grouped by medical class year and baseline characteristics of anti-anxiety medication users and non-users in November 2020

Item	Anxiety Level								
	Normal		Mild		Moderate		Severe		Total
	*N*	*%*	*N*	*%*	*N*	*%*	*N*	*%*	*N*
**Medical Year:**									
Preparatory Year	126	28.1	137	30.5	110	24.5	76	16.9	449
1st Year	233	21.4	333	30.6	320	29.4	203	18.6	1089
2nd Year	253	23.9	338	31.9	283	26.7	185	17.5	1059
3rd Year	315	25.8	368	30.2	310	25.4	227	18.6	1220
4th Clinical Year	391	30.3	427	33.0	283	21.9	191	14.8	1292
5th Clinical Year	506	34.6	451	30.9	312	21.4	192	13.1	1461
Internship Year	178	32.6	180	33.0	122	22.3	66	12.1	546
Total	2002	28.1	2234	31.4	1740	24.5	1140	16.0	7116
**Have you ever used antianxiety medications at any time during your college years?**									
No	1863	29.8	2017	32.3	1454	23.3	914	14.6	6248
Yes	139	16.0	217	25.0	286	32.9	226	26.0	868
Total	2002	28.1	2234	31.4	1740	24.5	1140	16.0	7116
**Which antianxiety medication have you used?**^a^									
Propranolol	75	19.0	98	24.9	125	31.7	96	24.4	394
Benzodiazepine	41	18.7	53	24.2	74	33.8	51	23.3	219
Antidepressants	99	30.0	102	30.9	86	26.1	43	13.0	330
Pregabalin	21	22.1	34	35.8	22	23.2	18	18.9	95

^a^*Respondents could select more than one answer*

### Perceived psychological stress during traditional versus online learning during the pandemic with its associated contributing factors

Participants reported higher stress levels during online learning (*M* = 1.83, *SD* = 1.244) compared to traditional teaching (*M* = 1.70, *SD* = 1.226). Pre-clinical students experienced more stress (*M* = 1.94, *SD* = 1.243) than clinical students (*M* = 1.71, *SD* = 1.233). Females experienced more stress than males (*M* = 2.10, *SD* = 1.234 .vs *M* = 1.56 *SD* = 1.195). In online teaching, levels of stress declined through the years, with first-years experiencing the highest levels of stress (*M* = 2.01, *SD* = 1.72), and fifth-years the lowest (*M* = 1.64, *SD* = 1.21) However, those in their internship year showed a slight increase in the level of stress (*M* = 1.66, *SD* = 1.40). A significant difference was found in female students, basic and clinical students, and non-users of anti-anxiety medications (*P* = <.0001, *P* = <.001, *P* = .003, *P* = <.0001, respectively). However, there was no significant difference between males and users of anti-anxiety medications (*P* = .157 and *P* = .192, respectively) (Table 2).

**Table 2:** A single-item measure of stress experienced since the shift to online learning and during traditional face-to-face learning, as reported by 7116 medical students across the Kingdom of Saudi Arabia in November 2020, sub-grouped by gender, medical class year, and medication usage

	Stress during online learning			Stress during traditional face to face learning		***P*** Value
	*N*	*M*	*SD*	*M*	*SD*	
**Gender**						
Female	3583	2.10	1.234	1.88	1.239	**< .0001**
Male	3533	1.56	1.195	1.52	1.185	.157
Total	7116	1.83	1.244	1.70	1.226	
**Basic Students**						**< .001**
Preparatory Year	449	1.87	1.282	1.60	1.225	
1st Year	1089	2.01	1.214	1.72	1.196	
2nd Year	1059	1.92	1.236	1.77	1.225	
3rd Year	1220	1.92	1.259	1.87	1.229	
Total	3817	1.94	1.243	1.77	1.220	
**Clinical Students**						**.003**
4th Year	1292	1.80	1.241	1.73	1.270	
5th Year	1461	1.64	1.214	1.61	1.207	
Internship Year	546	1.66	1.252	1.40	1.152	
Total	3299	1.71	1.233	1.62	1.228	
**Antianxiety Medication Use**						
No	6248	1.80	1.233	1.66	1.217	**< .0001**
Yes	868	2.06	1.300	1.98	1.257	.192
Total	7116	1.83	1.244	1.70	1.226	

**Fig. 2:**
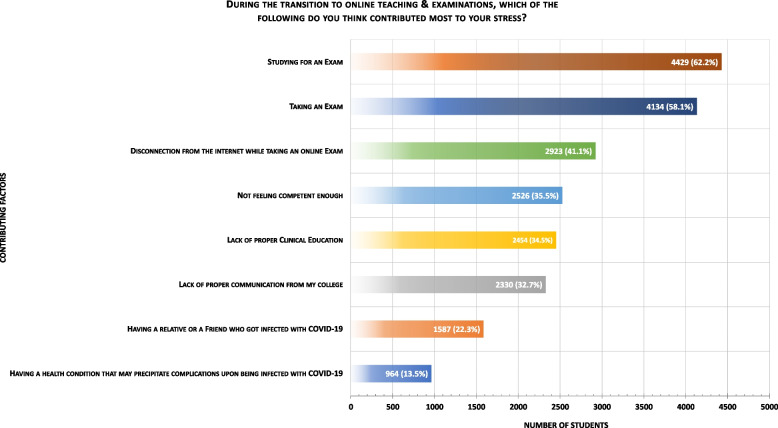
Factors contributing to stress imposed by the transition to online teaching and examinations reported by 7116 medical students across the Kingdom of Saudi Arabia in November 2020

Figure 2 illustrates the contributing factors that affected stress levels. Most students (62.2%, *n* = 4429) found that studying for an exam contributed the most to their stress levels, followed by taking an exam (58.1%, *n* = 4134), concerns about Internet connectivity (41.1%, *n* = 2923), feeling incompetent (35.5%, *n* = 2526), lack of proper clinical education (34.5%, *n* = 2454), lack of proper communication from college (32.7%, *n* = 2330), COVID-19 infection of a relative or friend (22.3%, *n* = 1587), and having a health condition that may precipitate complications upon being infected with COVID-19 (13.5%, *n* = 964).

Results for history and pattern of anti-anxiety medications usage are reported separately, [see Supplementary Fig. 1 and Table 2, Additional file 1.

Briefly among users of medications 40.4% (*n* = 351) students decreased their medication dose after switching to online learning, while 29% (*n* = 252) and 30.5% (*n* = 265) increased or maintained their doses, respectively. Students used medications mostly during final exams and before the objectively structured clinical examination (OSCE) or oral exam. These two activities necessitated the usage of higher doses than usual. About 45.8% (*n* = 397) either self-prescribed these medications or were taken through their colleagues.

### Coping strategies

**Fig. 3:**
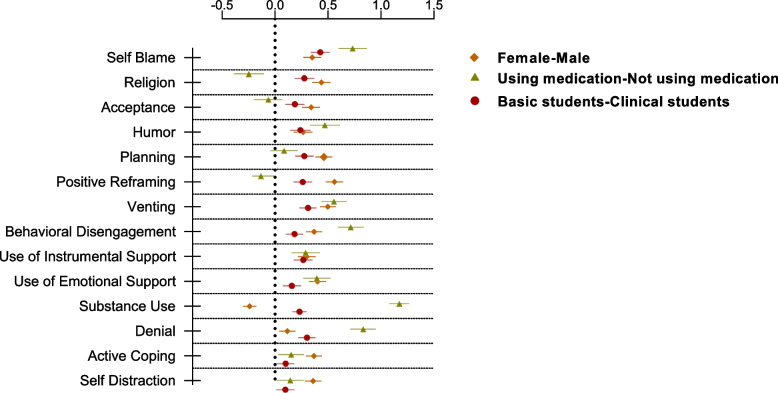
Linear regression estimates (with 95% CIs) of the differences in overall mean coping strategy values endorsed by 7116 medical students across the Kingdom of Saudi Arabia while controlling for gender, medication use, and medical class level in November 2020

We conducted a univariate linear regression to determine whether gender, medication usage, and student class year significantly predicted coping strategies. As shown in Fig. 3, female and basic year students reported using both positive and negative coping mechanisms more, except that females were much less likely to use substances compared to males. The figure also shows that medication usage was negatively associated with religion and positively associated with substance use, denial, self-blame, and behavioral disengagement.

## Discussion

Due to the current COVID-19 outbreak, the global prevalence of psychological disorders among medical students has emerged distinctly [7]. Psychological distress fueled by the demanding nature of medical education and the unprecedented global impact of the COVID-19 pandemic necessitates immediate intervention. The present study highlights the impact of the pandemic on the mental health of students from a large, socio-demographically heterogenous cohort in both public and private medical colleges. The current study also evaluated the national prevalence of anxiety and the subsequent use of medication or coping strategies among medical students during the COVID-19 outbreak. The GAD-7 scale was utilized in our study as a screening tool to assess the severity of anxiety among medical students [30].

### Anxiety levels among medical students

In the wake of the pandemic, nearly 60% of the students met the minimum anxiety score criteria. Notably, the survey was distributed in November 2020 during the pandemic period with strict curfew enforcement, which exacerbated the highly prevalent anxiety symptoms—nearly 40% of the medical students suffered from moderate to severe anxiety. The students’ year of study did not seem to influence the severity of anxiety. Our findings are consistent with national and international studies, as college students’ anxiety levels have generally increased during the COVID-19 outbreak [31–34], which has created a stressful studying environment. However, the levels of anxiety dropped significantly among medical students in their last 2 years of study (senior students), refuting our initial prediction (hypothesis 1). The observed decrease in anxiety levels might be attributed to limited clinical training and examination sittings. A previous study did not show statistically significant differences between clinical or pre-clinical undergraduate studies and psychological well-being [35]. However, our findings indicate that students in the clinical phase, versus those in the pre-clinical years, accounted for a higher proportion of students with no anxiety symptoms (32.6%, *n* = 1075 versus 24.3%, *n* = 927) or mild anxiety symptoms (32.1%, *n* = 1058 versus 30.8%, *n* = 1176). The claim that the transition to online teaching posed a challenging and stressful psychological situation has been confirmed by our results as students reported higher stress levels during online learning (*M* = 1.83, *SD* = 1.244) compared to traditional teaching (*M* = 1.70, *SD* = 1.226), lending credence to our prediction (hypothesis 2). The transition has indeed led to increased stress, especially in juniors and females. A similar observation was made by Abdulghani et al., where female students reported higher levels of stress than male peers [36]. Considering this, it is unjustified to extrapolate this increase in stress solely to online transition as the world was defenseless to the pandemic’s ripple effects, leaving medical students with extreme trepidation and uncertainty. Factors such as unprecedented solitude, missed academic opportunities, fogged career options, and fear for loved ones can lead to psychological suffering. Thus, efforts to manage external and internal stressors by implementing coping strategies are of great importance in achieving psychological well-being.

### Coping mechanisms adopted by medical students

In the current study, we evaluated whether gender, medication usage, and students’ year of study significantly predicted coping strategies using univariate linear regression among different cohorts of the surveyed students. Among them, male students reported more substance use as a coping strategy, whereas other coping strategies, regardless of their positive impact, were more pronounced in female students. Interestingly, a prior history of taking medications was associated with vulnerability to substance use, self-blame, behavioral disengagement, and lower levels of religious beliefs. Our results, however, suggest that clinical students evidently used coping strategies more than junior students. These findings suggest that clinical year students might have higher psychological resilience and better coping strategies [37]. The higher level of education and psychological maturity might play a role in minimizing anxiety levels among senior students [37]. As shown recently by Arima et al., self-efficacy and self-esteem are possibly other attributable motivational beliefs responsible for implementing positive coping strategies and improved resiliency [38]. Resiliency is positively associated with positive coping strategies such as positive reframing and acceptance. While our work did not categorize the positive or negative attributes of the coping strategies, individual coping strategies can be differentially evaluated. A previous 10-year longitudinal study conducted on a national scale suggested that implementing various coping strategies was usually indicative of a successful medical career.

### Anti-anxiety medications usage among medical students

Surprisingly, about 16% (*n* = 139) of the students used medication improperly, suggesting the studied group’s susceptibility to medication abuse. Half of the surveyed students used medications without prescription to deal with various stressful situations, including OSCE and oral presentations. These two academic activities appear to be the most common reasons for taking medication; however, it is important to acknowledge that these two activities are probably the major stressors in a student’s life regardless of the pandemic or the academic environment. One unanticipated finding supporting our prediction (hypothesis 3) was that anti-anxiety medication usage during the switch to online learning decreased by 40% (*n* = 351). Nevertheless, the studied group of anti-anxiety medication users (*n* = 868) still reported higher stress during the transition to online learning compared to traditional face-to-face teaching (*M* = 2.06, *SD* = 1.3 versus *M* = 1.98, *SD* = 1.25). This observed decrease in medication usage might be attributed to the fact that students were not under the same circumstances in which certain academic activities were conducted. It can thus be suggested that transitioning to online learning offered some students an environment in which they no longer felt compelled to use anti-anxiety medications. However, the observed difference between stress during online and traditional face-to-face learning among users of anti-anxiety medications in this study was not statistically significant (*P* = .192). These findings might not be representative since we only utilized a single-item measure of stress, which has not yet been validated in areas other than work-related organizational studies [27]. Along those lines, the largest proportion of studied medication was propranolol, which is often prescribed off-label to treat anxiety. Notably, there is insufficient evidence to support its anxiolytic effect as a form of medication [39]. Two recent national studies have suggested the prevalence of propranolol usage among medical students [21, 40]. The current study highlights that nearly half of the medication users have used it as a treatment option. Hence, it can be concluded that several students take poorly suited medications for a potential undiagnosed underlying anxiety disorder.

### Factors contributing to stress imposed by the transition to online teaching and examinations

Prior to the COVID-19 pandemic, poor exam preparation, framework, and organizational structure of medical education were considered a substantial source of psychological distress [41]. As shown by the present study, contributing factors underpinning the challenges of the sudden transition are exam preparation and subsequently, the online exam experience with possible Internet connection problems. In addition, the lack of proper communication from the college, insufficient clinical education, a relative or a friend contracting COVID-19 infection, or the fear of getting infected by the virus were factors contributing to stress. These results are in line with recent studies which found that the fear of infection, social isolation, and uncertainty of returning to normal life are among the most common factors impacting the mental health of medical students [34, 41]. Of note, our study did not investigate concerns related to social isolation as most medical students in the kingdom of Saudi Arabia live at home with their families.

### Implications of the study’s findings

The findings of this study can provide theoretical evidence for implementing supportive psychological assistance and guidelines for leaders of academic institutes globally to overcome the potential deterioration of academic excellence.

Additionally, it seems imperative to inaugurate personalized workshops for medical student groups with significant emphasis on strategies for coping with stress; concurrently, it is also pivotal to enforce coping skills courses into the medical curriculum, especially for junior students, thereby building their resilience and resourcefulness. Furthermore, it is crucial to introduce specialized lectures on the use and abuse of therapeutic agents for anxiety and stress in pharmacology and psychology courses while urging the relevant authorities to effectuate regulations to reinforce prescriptions for propranolol.

The role of medical school counselors should be implemented, and the personal experiences of senior students should be incorporated throughout the design of preventive measures since they possess better active and adaptive coping strategies, as suggested by the present study. Student-to-students support groups should be encouraged to help overcome avolition.

Although the pandemic compelled students to migrate to online remote teaching and learning, throughout these unique experiences, it opened possible doors for a potential online hybrid model without compromising the medical education process of learning while improving student-centeredness and boosting virtual mentorship platforms.

## Limitations

This study has some limitations. Our study was based on a self-administered online survey that can be subjectively perceived. Furthermore, our study utilized self-report measures, which might introduce recall bias that could potentially overestimate or underestimate the study’s findings. Stigma may also have contributed to underreporting of psychological symptoms. Our findings captured a period of great stress worldwide; yet historically, looking at one’s stress pre-pandemic might not be the best indicator of pre- or post-pandemic, as there could be a possibility for both overreporting and underreporting. Additionally, the potential cultural impacts and ethnic diversity might impose difficulties related to generalizability for all medical school cohorts. For instance, substance use may be resorted to by higher percentages elsewhere. Also, our study did not investigate concerns related to social isolation as most medical students in Saudi Arabia live at home with their families, which is uncustomary abroad. Another limitation was our use of cross-sectional design, which limited our ability to make inferences about causality related to the impact of COVID-19. Finally, we used a convenience sampling technique, limiting the generalizability of this study’s findings.

## Conclusion

The results of the current study provide a national overview of the impact of COVID-19 on the mental health of medical students. We aimed to expand a huge body of evidence on the psychological distress associated with the current public health crisis and prepare for similar unforeseen catastrophic situations. Preparing for the negative impact of such adverse situations can be useful in minimizing serious psychological complications and enabling a successful medical career. This can be achieved by increasing the awareness of mental health disorders through stress management campaigns, the de-stigmatization of mental health illnesses, and the provision of optimal psychological support services.

Our study was based on a relatively large sample of socio-demographically heterogenous medical students from both public and private universities in rural and urban areas throughout Saudi Arabia. Therefore, this study can establish a conceptual framework to minimize and cushion any upcoming challenges that deteriorate the mental health of medical students worldwide. Despite the exciting results, global meta-analysis studies can provide more supplemental and useful information on how to attain an optimal medical and educational environment. Further research should also be undertaken to investigate whether the immunity gained by the public through vaccine campaigns might lead to different perceptions of the pandemic’s effect on the mental health of medical students.

## Supplementary Information

**Additional file 1: Appendix 1 Supplementary Table 1.** Names of all 38 medical colleges (government and private) across all regions of Saudi Arabia and the number of students who participated in the study from each university (*N* = 7116). **Supplementary Fig. 1.** The effects of online learning on medication usage reported by 868 medical students across the Kingdom of Saudi Arabia. **Supplementary Table 2.** Baseline characteristics and patterns of medication usage among 868 medical students across the Kingdom of Saudi Arabia.

## Data Availability

The datasets used and/or analysed during the current study are available from the corresponding author on reasonable request.

## Abbreviations

CIs: Confidence intervals
GAD-7: Generalized anxiety disorder
IRB: Institutional Review Board
MOH: Ministry of Health
OSCE: Objectively structured clinical examination
SPSS: Statistical Package for the Social Sciences
