# Supplementary material for: Prevalence of pharmacological and non-pharmacological coping mechanisms for anxiety management during the COVID-19 pandemic: investigating the transition to online learning among medical students
Source: BMC Psychiatry. 2022 Nov 14;22:704. doi: 10.1186/s12888-022-04372-6 (PMC9662775; doi:10.1186/s12888-022-04372-6)
Supplement: Supplementary file 1 — Additional file 1: Appendix 1 Supplementary Table 1. Names of all 38 medical colleges (government and private) across all regions of Saudi Arabia and the number of students who participated in the study from each university (N = 7116). Supplementary Fig. 1. The effects of online learning on medication usage reported by 868 medical students across the Kingdom of Saudi Arabia. Supplementary Table 2. Baseline characteristics and patterns of medication usage among 868 medical students across the Kingdom of Saudi Arabia. [file 12888_2022_4372_MOESM1_ESM.docx]

**Additional file 1**

**Supplementary Table 1**

Names of all 38 medical colleges (government and private) across all regions of Saudi Arabia and the number of students who participated in the study from each university (N = 7,116)

| **Regions** | **N** | **%** |
| --- | --- | --- |
| **Central Region:** |  |  |
| -1 Imam Muhammad Bin Saud Islamic University College of Medicine - Riyadh | 170 | 2.4 |
| -2 Almaarefa Medical College - Riyadh | 167 | 2.3 |
| -3 Almajmaah University College of Medicine - Al Majma'ah | 132 | 1.9 |
| -4 Alfaisal University College of Medicine - Riyadh | 333 | 4.7 |
| -5 College of Medicine of Alfarabi Private Colleges - Riyadh | 165 | 2.3 |
| -6 Dar Al Uloom University College of Medicine - Riyadh | 241 | 3.4 |
| -7 King Saud University, College of Medicine - Riyadh | 204 | 2.9 |
| -8 King Saud bin Abdulaziz University for Health Sciences College of Medicine - Riyadh | 275 | 3.9 |
| 9- Prince Sattam bin Abdulaziz University College of Medicine - Al Kharj | 87 | 1.2 |
| 10- Princess Nourah Bint Abdulrahman University College of Medicine – Riyadh | 124 | 1.7 |
| 11- Shaqra College of Medicine - Shaqra | 57 | .8 |
| 12- Shaqra College of Medicine in Dawadmi - Al- Dawadmi | 90 | 1.3 |
| 13- Sulaiman Alrajhi College of Medicine - Al Bukairyah | 133 | 1.9 |
| 14- Qassim University College of Medicine - Burayda | 198 | 2.8 |
| 15- Unaizah College of Medicine and Medical Sciences, Qassim University - Unaizah | 179 | 2.5 |
| **Eastern Region:** |  |  |
| 16- Imam Abdulrahman Bin Faisal University College of Medicine - Dammam | 197 | 2.8 |
| 17- King Faisal University, College of Medicine - Al-Ahsa | 264 | 3.7 |
| **Western Region:** |  |  |
| 18- Al Rayan Medical College - Madinah al munawarah | 178 | 2.5 |
| 19- Batterjee Medical College for Sciences and Technology - Jeddah | 159 | 2.2 |
| 20- King Saud bin Abdulaziz University for Health Sciences College of Medicine - Jeddah | 266 | 3.7 |
| 21- King Abdulaziz University - College of Medicine in Jeddah | 170 | 2.4 |
| 22- Umm Al-Qura University - College of Medicine in Mecca - Mecca | 282 | 4.0 |
| 23- University of Jeddah Faculty of Medicine - Jeddah | 174 | 2.4 |
| 24- Fakeeh College for Medical Sciences - Jeddah | 61 | .9 |
| 25- College of Medicine in Al-Qunfudah - Umm Al-Qura University - Al-Qunfudah | 281 | 3.9 |
| 26- Ibn Sina National College for Medical Studies Faculty of Medicine - Jeddah | 261 | 3.7 |
| 27- King Abdulaziz University - College of Medicine in Rabigh (Rabigh Branch) | 203 | 2.9 |
| 28- Taibah University College of Medicine - Madinah al munawarah | 236 | 3.3 |
| 29- Taif University College of Medicine - Taif | 185 | 2.6 |
| **Southern Region:** |  |  |
| 30- Al Baha University School of Medicine - Al Baha | 239 | 3.4 |
| 31- Jazan University Faculty of Medicine - Jazan | 194 | 2.7 |
| 32- King Khalid University College of Medicine and Health Sciences - Abha | 174 | 2.4 |
| 33- University of Bisha College of Medicine - Bisha | 91 | 1.3 |
| 34- Najran University College of Medicine - Najran | 211 | 3.0 |
| **Northern Region:** |  |  |
| 35- Al Jouf University College of Medicine - Al Jouf | 246 | 3.4 |
| 36- Northern Borders University College of Medicine - Arar | 103 | 1.4 |
| 37- University of Tabuk Faculty of Medicine - Tabuk | 185 | 2.6 |
| 38- University of Hail College of Medicine - Hail | 201 | 2.8 |
| **Total:** | 7116 | 100 |

**Supplementary Figure 1**

The effects of online learning on medication usage reported by 868 medical students across the Kingdom of Saudi Arabia


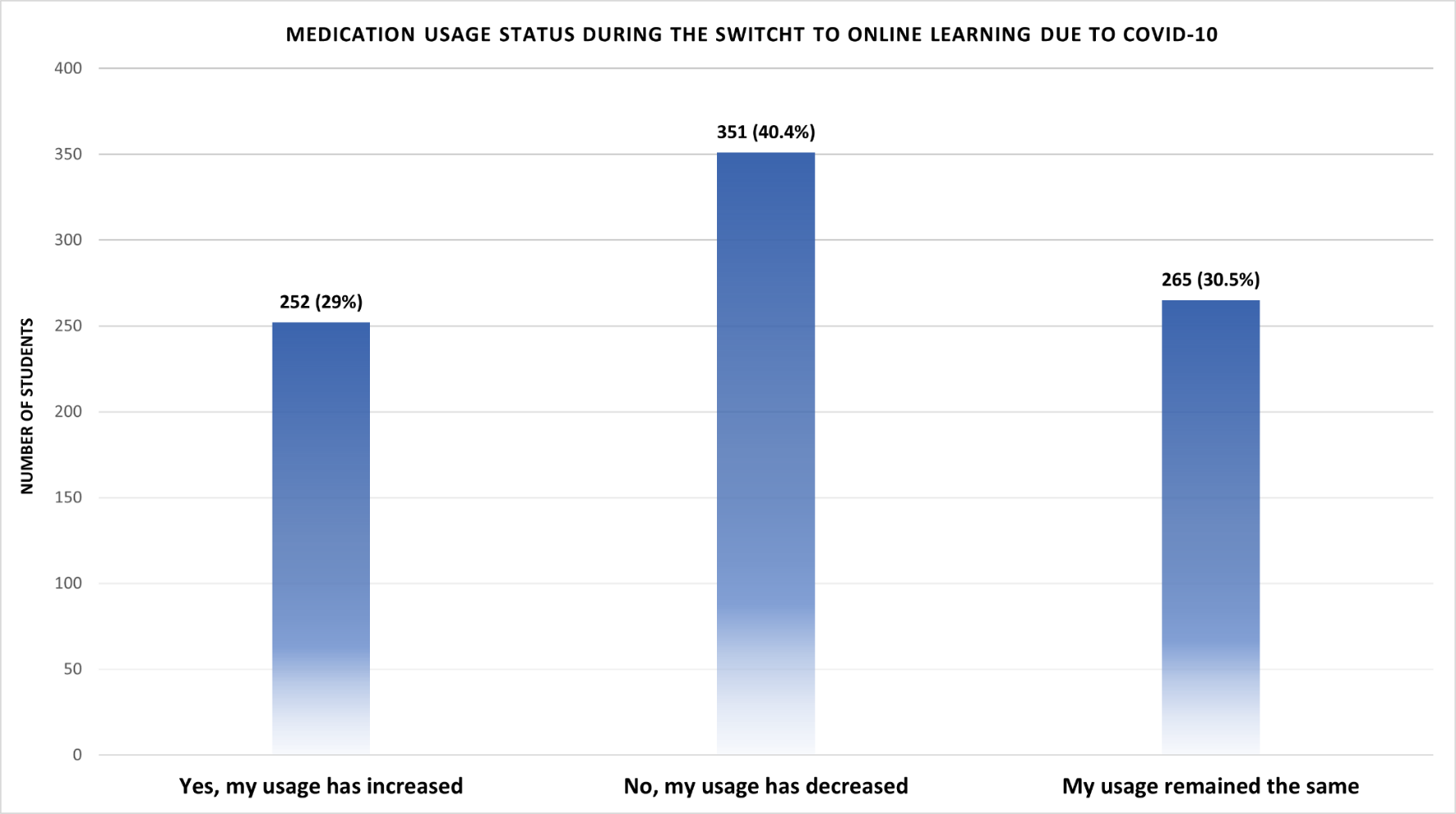


**Supplementary Table 2**

Baseline characteristics and patterns of medication usage among 868 medical students across the Kingdom of Saudi Arabia

| Item |  | **Number** | **Percentage (%)** |
| --- | --- | --- | --- |
| **When do you use any of anti-anxiety medications the most?**^a^ | | | |
| Before oral exams (OSCE) |  | 308 | 35.5 |
| Before poster or oral presentations |  | 263 | 30.3 |
| During final exams |  | 311 | 35.8 |
| During mid-term exams period |  | 195 | 22.5 |
| **What are the reasons to use any of these anti-anxiety medications?**^a^ | | | |
| For medical reasons |  | 382 | 44.0 |
| To relief anxiety |  | 499 | 57.5 |
| Peer pressure |  | 154 | 17.7 |
| **Who prescribed any of these anti-anxiety medications for you?** | | | |
| Yourself |  | 234 | 27.0 |
| Your colleague |  | 163 | 18.8 |
| Your physician |  | 455 | 52.4 |
| **What is your usual dose of any of these anti-anxiety medications?** | | | |
| I use less than the recommended dose |  | 192 | 22.1 |
| I use the recommended dose |  | 434 | 50.0 |
| I use more than the recommended dose |  | 173 | 19.9 |
| I don't know |  | 69 | 7.9 |
| **What activity requires you to use higher than your usual dose?** ^a^ | | | |
| Before oral exams (OSCE) |  | 138 | 15.9 |
| Before poster or oral presentations |  | 123 | 14.2 |
| During final exams |  | 168 | 19.4 |
| During mid-term exams |  | 129 | 14.9 |
| I do not increase my dose |  | 445 | 51.3 |
| **Have you noticed that you need to increase the dose over time?** | | | |
| No |  | 395 | 45.5 |
| Yes |  | 473 | 54.5 |

^a^ *Respondents could select more than one answer*
